# Clinical significance of intronic variants in BRAF inhibitor resistant melanomas with altered *BRAF* transcript splicing

**DOI:** 10.1186/s40364-017-0098-3

**Published:** 2017-05-11

**Authors:** Gulietta M. Pupo, Suzanah C. Boyd, Carina Fung, Matteo S. Carlino, Alexander M. Menzies, Bernadette Pedersen, Peter Johansson, Nicholas K. Hayward, Richard F. Kefford, Richard A. Scolyer, Georgina V. Long, Helen Rizos

**Affiliations:** 1 Centre for Cancer Research, The Westmead Millennium Institute for Medical Research, University of Sydney, Westmead Hospital, Westmead, NSW Australia; 2 Faculty of Medicine and Health Sciences, Macquarie University, Sydney, NSW Australia; 3 Melanoma Institute Australia, Sydney, NSW Australia; 4 Departments of Medical Oncology, Crown Princess Mary Cancer Centre, Westmead Hospital, Sydney, NSW Australia; 5 Disciplines of Medicine, Sydney Medical School, The University of Sydney, Sydney, NSW Australia; 6 Disciplines of Pathology, Sydney Medical School, The University of Sydney, Sydney, NSW Australia; 7 Oncogenomics Laboratory, QIMR Berghofer Medical Research Institute, Herston, Brisbane, QLD Australia; 8 Departments of Tissue Pathology and Diagnostic Oncology Royal Prince Alfred Hospital, Camperdown, NSW Australia

**Keywords:** Melanoma, BRAF inhibitor, BRAF splicing, Dabrafenib, Resistance

## Abstract

Alternate BRAF splicing is the most common mechanism of acquired resistance to BRAF inhibitor treatment in melanoma. Recently, alternate BRAF exon 4–8 splicing was shown to involve an intronic mutation, located 51 nucleotides upstream of *BRAF* exon 9 within a predicted splicing branch point. This intronic mutation was identified in a single cell line but has not been examined in vivo. Herein we demonstrate that in three melanomas biopsied from patients with acquired resistance to BRAF inhibitors, alternate BRAF exon 4–8 splicing is not associated with this intronic branch point mutation. We also confirm that melanoma cells expressing BRAF splicing variants retain exquisite sensitivity to existing FDA-approved MEK inhibitors.

**Electronic supplementary material:**

The online version of this article (doi:10.1186/s40364-017-0098-3) contains supplementary material, which is available to authorized users.

## Main text

The serine/threonine kinase BRAF is mutated and constitutively activated in 40–60% of cutaneous melanomas. Selective inhibitors of mutant BRAF, including dabrafenib and vemurafenib, improve the progression-free and overall survival of BRAF-mutant melanoma patients [1, 2]. Despite this activity, acquired resistance develops in most melanoma patients and multiple mechanisms of resistance have been described. Most of these resistance effectors, including alterations in MEK1, BRAF and N-RAS, promote the reactivation of the mitogen activated protein kinase pathway [3]. Alternate splicing of oncogenic BRAF is the most common driver of acquired resistance to BRAF inhibitors and is evident in approximately 30% of resistant melanomas [4–6]. Splice variants of BRAF encode an active kinase, but lack an intact RAS binding domain. These truncated proteins are prone to dimerization and have reduced affinity for the class I BRAF inhibitors, vemurafenib and dabrafenib [4]. Predictably, melanomas expressing BRAF splice variants display MAPK re-activation in the presence of BRAF inhibitors, and retain sensitivity to the inhibition of the downstream kinases MEK and ERK [5, 7].

One mechanism of altered BRAF splicing was recently shown to involve a C-to-G mutation in intron 8 within a predicted splicing branch point, 51 nucleotides upstream of BRAF exon 9 (i.e C > G − 51). This intron 8, variant was sufficient to promote the expression of a BRAF transcript lacking exons 4–8 (BRAF exon 4–8∆; also referred to as BRAF3–9) in a single vemurafenib resistant melanoma cell line [8]. The significance of this intronic mutation was not clinically validated, although we are aware of five patients with acquired BRAF inhibitor resistance driven by the BRAF exon 4–8∆ alternate transcript [4–6].

**Fig. 1:**
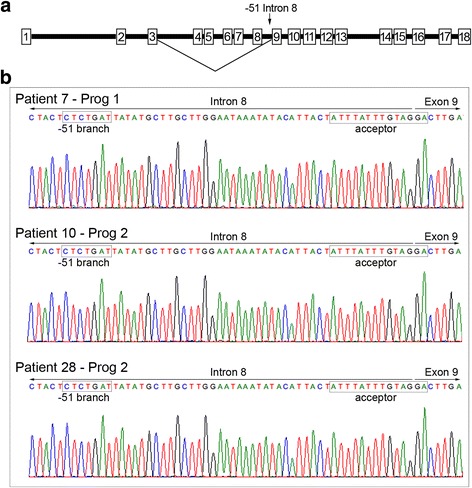
*BRAF* intronic splice and branch point sequences in melanomas expressing the BRAF exon 4–8∆ transcript. **a** Schematic diagram of the *BRAF* gene, showing exons (*numbered boxes*) and introns (*black bars*) and the alternate splicing event leading to the BRAF exon 4–8∆ transcript. **b** Sanger sequencing traces showing the sequence encompassing the junction between BRAF intron 8 and exon 9 in BRAF inhibitor resistant melanomas (Progs) expressing the BRAF exon 4–8∆ transcripts and derived from patient 7, 10 and 28. The branch point motifs, including the −51 nucleotide associated with BRAF exon 4–8∆ splicing, and splice sites are *boxed*

We selected three of these five BRAF inhibitor resistant patients (Patients 7, 10 and 28 [5]). These BRAF^V600^-mutant patients progressed within 1 year of receiving BRAF inhibitor monotherapy and analysis of the matched pre-treatment and progressing tumours revealed that only the progressing biopsies expressed the BRAF exon 4–8∆ splice variant (Additional file 1: Figure S1). We analysed the intron 8 splicing branch site in these matched pre-treatment and progressing melanomas and in all melanomas the -51– -45 branch point sequence and the exon 9 acceptor splice site were wild type (Fig. 1).

We analysed the impact of the C > G − 51 intron 8 mutation using several algorithms, and it was not predicted to break the branch point nor alter splice factor binding (Table 1). The −51 G mutation potentially activates an intronic cryptic donor site (Table 1), but usage of this splice site would not generate the BRAF exon 4–8∆ transcript. Moreover, an alignment of 66 intronic branch point disease mutations revealed that mutations altering splicing occur predominantly at the highly conserved adenine residue (equivalent to −48 in the intron 8 branch site), with no reported disease causing mutation at the position equivalent to −51 [9]. It is also worth noting that unlike the branch point adenine, which is highly conserved in mammalian branch point sequences, the −5 branch point nucleotide (equivalent to the −51 BRAF intron 8 branch site) is degenerate in mammals [10], and the BRAF intron 8 sequence shows a cytosine to thymine bias in many non-human primates (data not shown).

**Table 1:** Predicted effects of −51 mutation in intron 8

	Branch Site	Donor Splice	SRSF6
	Position/Site/Score	Position/Site/Score	Sequence/Site/Score
Wild Type	-51/CTCTGAT/88	-54/ACTctcgat/40	-134/TGTGTA/84
Mutant	-51/**G**TCTGAT/86	-138/ACTgtctga/67	-134/TGTGTA/84

Data derived using the Human Splice Finder Tool [12]

Branch Site score above 67 is considered a potential break point, and score variation between wild type and mutant sequence of less than −10% is considered to break the branch point

Splice Site values above 65 are predicted splice sites, the −51 G mutation is underlined in Cryptic Donor Site

Splicing factor SRSF6 motif with highest predicted scores is shown [12]

Motif position is relative to first nucleotide of exon 9

Significantly, the expression of BRAF splice variants is insufficient to confer MEK inhibitor resistance in melanoma (Additional file 2: Figure S2). Consequently, BRAF^V600^-mutant metastatic melanoma patients treated with combination BRAF and MEK inhibitors, rarely develop resistance due to alternate BRAF splicing [11]. In this clinical setting, therefore, the therapeutic potential of splicing modulators, such as spliceostatin A, which inhibit the formation of the BRAF exon 4–8∆ via inhibition of the SF3B1 splicing factor [8], is diminished.

BRAF inhibitor resistance mediated by alternate BRAF splicing is likely a result of *cis*-acting alterations that disrupt the use of allele-specific constitutive splice sites. *Trans*-acting mutations can also affect basal and alternate splicing machinery, but these mutations would be predicted to alter the processing of both wild type and mutant BRAF pre-mRNA. Several studies have now confirmed that the wild type *BRAF* allele is transcribed and processed correctly in mutant BRAF melanoma, and the shorter, variant *BRAF* transcripts are derived only from the mutant *BRAF* allele [4, 5]. Our data show that alternate BRAF exon 4–8∆ splicing in melanomas derived from patients with resistance to BRAF inhibitor monotherapy is not associated with a mutation in the −51 position in intron 8. Regardless of the precise mechanisms altering the splicing of BRAF, downstream MEK and ERK inhibition effectively inhibit the proliferation of BRAF inhibitor resistant melanoma cells expressing BRAF splice variants [5, 7].

## Methods

### Patients

All patients included in this study had BRAF^V600^ mutant metastatic melanoma, and were treated with either dabrafenib or vemurafenib [5]. All patients had a pre-treatment melanoma tissue sample obtained before commencing BRAF inhibitor and a matched progressing melanoma metastasis (Prog). Informed consent was obtained for each patient under approved Human Research Ethics Committee protocols.

### BRAF sequence analysis

Sequences were amplified using *Taq* polymerase (Fisher Biotech) using primers shown in Additional file 3: Table S1. PCR products were purified using QIAquick PCR purification kit (Qiagen, Limburg, Netherlands) followed by Sanger sequencing on the 3730xl DNA Analyser (AGRF, Westmead, NSW, Australia). The Human Splicing Finder system [12] was used to identify and analyse BRAF splice sites and branch point sites and auxiliary splicing enhancer sequences.

### Melanoma cell lines

The short-term patient-derived dabrafenib-resistant melanoma cells, WMD009 and SMU027, as well as SKMel28 parental and the dabrafenib-resistant BR4 cell line [7] were grown in in Dulbecco’s Modified Eagle Medium (DMEM) with 10% FBS and glutamine (Gibco-BRL).

## Additional files

Additional file 1: Figure S1. Sequence and expression of *BRAF* exon 4-8Δ splice variant in BRAF inhibitor resistant melanomas. A. PCR analysis of *BRAF* cDNA from pre-treatment (Pre) and matching melanomas progressing (Prog) on BRAF inhibitor monotheraphy. kb, kilobase; Lane 1 1 kb marker. B. Traces showing alternate *BRAF* splice junctions and the exon 15 Val600 codon (boxed) in BRAF inhibitor progressing melanomas expressing the *BRAF* exon 4-8Δ splice variant. (TIFF 676 kb)

Additional file 2: Figure S2. BRAF inhibitor-resistant melanoma cells expressing BRAF splice variants retain sensitivity to the FDA-approved MEK inhibitor, trametinib. Viability curves of the parental SKMel28 and the dabrafenib-acquired resistant BR4 subline, and patient-derived dabrafenib-resistant WMD009 and SMU027 cells treated with the indicated doses of dabrafenib or trametinib for 72 h (relative to DMSO-treated controls; mean ± SD; *n* = 2). (TIFF 411 kb)

Additional file 3: Table S1. Amplification and sequencing primers. (PDF 91 kb)
