# Supplementary figures and images for: Clinical significance of intronic variants in BRAF inhibitor resistant melanomas with altered BRAF transcript splicing
Source: Biomark Res. 2017 May 11;5:17. doi: 10.1186/s40364-017-0098-3 (PMC5426037; doi:10.1186/s40364-017-0098-3)

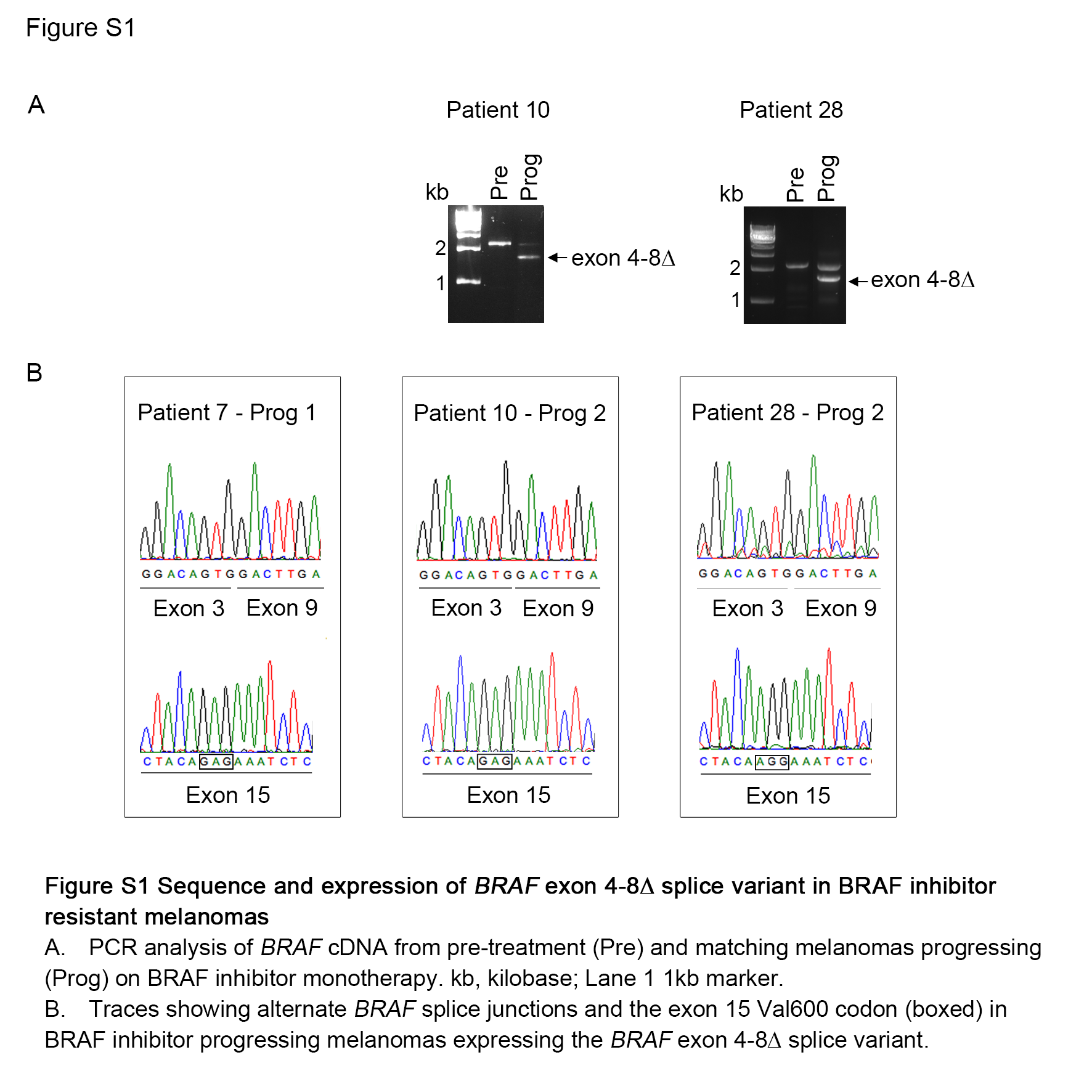

Supplement: Supplementary file 1 — Sequence and expression of BRAF exon 4-8Δ splice variant in BRAF inhibitor resistant melanomas. A. PCR analysis of BRAF cDNA from pre-treatment (Pre) and matching melanomas progressing (Prog) on BRAF inhibitor monotheraphy. kb, kilobase; Lane 1 1 kb marker. B. Traces showing alternate BRAF splice junctions and the exon 15 Val600 codon (boxed) in BRAF inhibitor progressing melanomas expressing the BRAF exon 4-8Δ splice variant. (TIFF 676 kb) [file 40364_2017_98_MOESM1_ESM.tif]

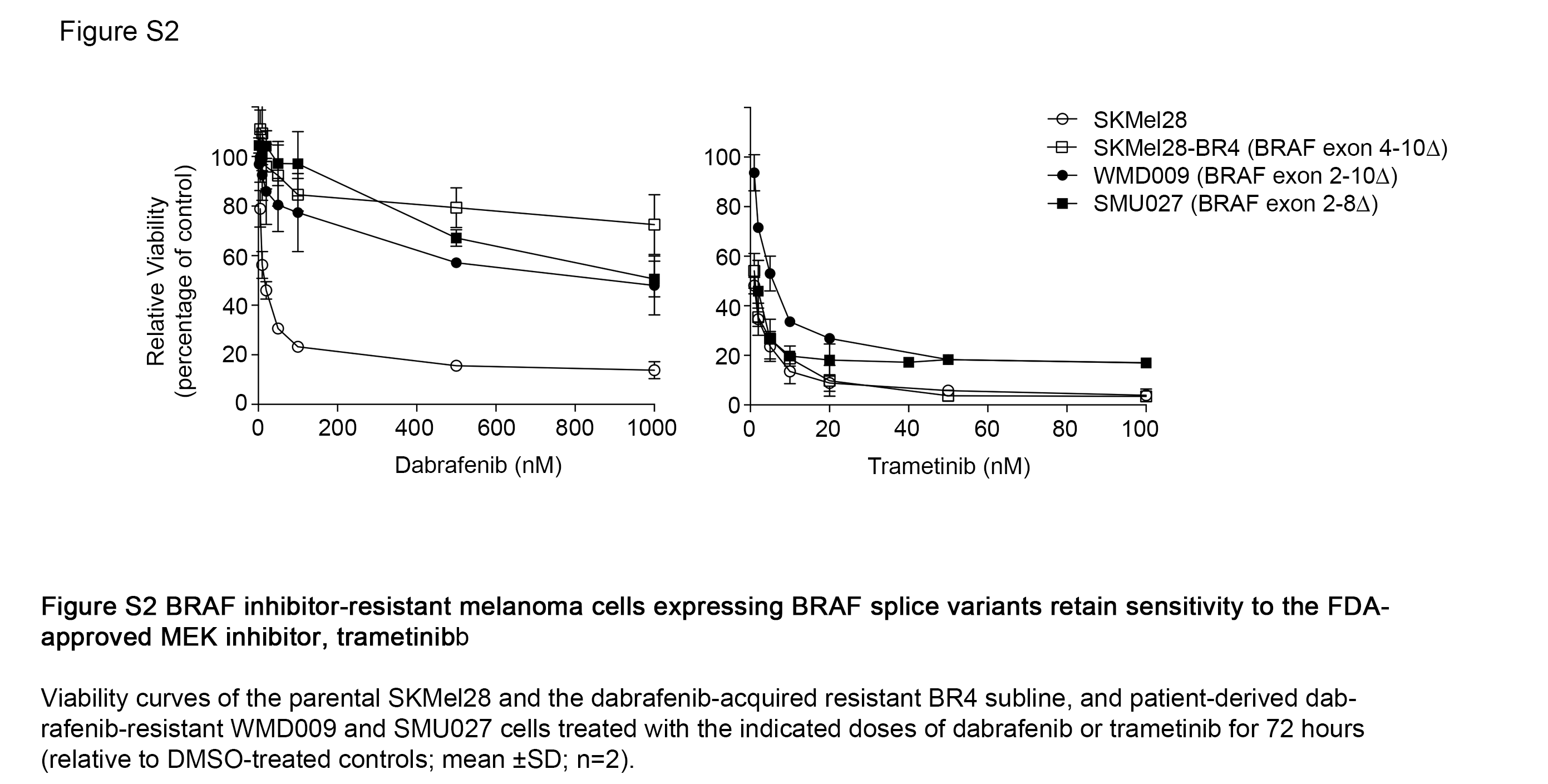

Supplement: Supplementary file 2 — BRAF inhibitor-resistant melanoma cells expressing BRAF splice variants retain sensitivity to the FDA-approved MEK inhibitor, trametinib. Viability curves of the parental SKMel28 and the dabrafenib-acquired resistant BR4 subline, and patient-derived dabrafenib-resistant WMD009 and SMU027 cells treated with the indicated doses of dabrafenib or trametinib for 72 h (relative to DMSO-treated controls; mean ± SD; n = 2). (TIFF 411 kb) [file 40364_2017_98_MOESM2_ESM.tif]
