# Supplementary material for: Clinical significance of intronic variants in BRAF inhibitor resistant melanomas with altered BRAF transcript splicing
Source: Biomark Res. 2017 May 11;5:17. doi: 10.1186/s40364-017-0098-3 (PMC5426037; doi:10.1186/s40364-017-0098-3)
Supplement: Supplementary file 3 — Amplification and sequencing primers. (PDF 91 kb) [file 40364_2017_98_MOESM3_ESM.pdf]

## Table S1. Amplification and Sequencing Primers

| | Forward | Reverse |
|---|---|---|
| **Exon3/Intron3** | TATGAAGAATACACCAGCAA | CAAGTTTGGCAGACAGGTTT |
| **Intron3/Exon4** | TGTGATTGAAGGGTTATACAG | ATCCTGAATTCTGTAAACA |
| **Intron8/exon9** | GGAAGCCATTGGTTTTGAAT | CTGTGTCTGTTACTTGAAAG |
| **Intron1/Exon2** | AGTATTTAATTATATGTGTACA | TCCAGATATATTGATGGTGGAT |
| **Exon1/Intron1** | GGCTCTCGGTTATAAGATGGC | GCTCAACCACCGCTGCCCCAAT |
| **BRAF cDNA** | GGCTCTCGGTTATAAGATGGC | ACAGGAAACGCACCATATCC |
